# Multiomics Revealed the Multi-Dimensional Effects of Late Sleep on Gut Microbiota and Metabolites in Children in Northwest China

**DOI:** 10.3390/nu15204315

**Published:** 2023-10-10

**Authors:** Xuesong Xiang, Juanjuan Chen, Mingyu Zhu, Huiyu Gao, Xiaobing Liu, Qi Wang

**Affiliations:** 1 Key Laboratory of Trace Element Nutrition of National Health Commission, National Institute for Nutrition and Health, Chinese Center for Disease Control and Prevention, Beijing 100050, China; xiangxs@ninh.chinacdc.cn (X.X.);; 2 Cuiying Biomedical Research Center, Lanzhou University Second Hospital, Lanzhou 730030, China

**Keywords:** children with late bedtime, gut microbiota and metabolites, plasma metabolites, *Akkermansia muciniphila*

## Abstract

Background Sleep plays a pivotal role in children’s mental and physical development and has been linked to the gut microbiota in animals and adults. However, the characteristics of the gut microbiota and metabolites and the relationship to late bedtimes in children remain unclear. Methods In total, 88 eligible children, aged from 3 to 8 years, were recruited and divided into two groups according to the bedtime collected by designed questionnaires (early, before 22:00: n = 48; late, after 22:00, n = 40). Stools and plasma samples were collected to examine the characteristics of the gut microbiota and metabolites by shotgun metagenomics and metabolomics. Results The richness and diversity of the gut microbiota in children with early bedtime were significantly increased compared with the late ones. *Coprococcus*, *Collinsella*, *Akkermansia muciniphila*, and *Bifidobacterium adolescentis* were significantly more abundant in children with early bedtime, while *Bacteroides* and *Clostridium* sp. CAG-253 were obviously enriched in the late ones. A total of 106 metabolic pathways, including biosynthesis of ribonucleotide, peptidoglycan, and amino acids, and starch degradation were enriched in children with early bedtime, while 42 pathways were abundant in those with late bedtime. Notably, more gut microbial metabolites were observed in children with late bedtime, which included aldehyde, ketones, esters, amino acids and their metabolites, benzene and substituted derivatives, bile acids, heterocyclic compounds, nucleotide and metabolites, organic acid and derivatives, sugars and acyl carnitine. In plasma, fatty amides, lipids, amino acids, metabolites, hormones, and related compounds were enriched in children with early bedtime, while bile acids were higher in children with late bedtime. Association studies revealed that the different microbial species were correlated with metabolites from gut microbiota and plasma. Conclusions The results of our study revealed that the gut microbiota diversity and richness, and metabolic pathways were significantly extensive in children with early bedtime, whereas the gut microbial metabolites were significantly decreased, which might be related to gut microbial differences.

## 1. Introduction

Sleep is essential and complex as a physiological activity, which occupies nearly one-third of the human life. The importance of sleep is increasingly recognized, but unhealthy sleep patterns, such as willful sleep deprivation and sleep disorders, are becoming widespread, and has caused a variety of serious health concerns in modern society [1,2]. Until now, a great many burdens for physical and psychological states owing to poor sleep are closely related to multiple health hazards [3], such as cardiovascular diseases, obesity, diabetes, and death. Nevertheless, some potential health benefits of sleep remain to be discovered, as are some functions and mechanisms of sleep. Circadian rhythms are endogenous and entrainable biological processes that regulate numerous biological functions and are impacted by changes in activity. Notably, shift work, staying up late, nighttime social activities, anxiety, and stress lead to sleep-awake disorders and disruption of circadian rhythms. In contrast to the effects of reduced duration of sleep, impacts of the time of day at which sleep starts are more prominent and might play a role in the development of obesity, neurocognitive dysfunction, and reduced academic performance and social adjustment.

In recent years, the multifaceted effects of gut microbiota on human health has been reported [4,5]. The microbiota-gut-brain axis involved in the regulation of sleep has an irreplaceable role in the etiology and pathogenesis of sleep disorders. Furthermore, the perturbation of the gut microbiota and metabolites have been demonstrated in modulating multiple physiological functions, such as energy metabolism, immune defense, downstream effects on circadian pattern [4]. Abundant research of the complex interplay between sleep behavior and gut microbiota has been sequentially reported in animal and adults [6,7]. The composition of the gut microbiota, i.e., the variety and relative abundance of bacterial taxa in the gastrointestinal system, could affect sleep directly or indirectly through a wide range of sleep-related substances. Moreover, sleep behavior could be correlated with the gut microbiota by a bidirectional communication between the gut and the brain [7,8].

To date, growing evidence shows that appropriate sleep timing is a significant determinant for normal growth and development in children. Childhood is recognized as a key period during which sleep, the gut microbiota, and physical development are gradually transitioning to adult-like patterns [9]. Some studies recently suggested that late sleep and propensity for late evening activities could be linked to overweight and obesity in children. Regrettably, few studies investigated the complicated relationship of sleep behavior, the gut microbiota, gut microbial and plasma metabolites. Similar studies are still limited to children in China, although the increasing general trend of late sleep in children is concerning. The current study on children living in northwest China could provide valuable information on the complicated relationships and possible mechanisms associated with late sleep from many perspectives, using multi-omics data. Moreover, this study would provide the theoretical reference to improve sleep habits for those in education and health services.

## 2. Materials and Methods

### 2.1. Subjects and Study Design

This study was performed on pediatric outpatients and aimed to examine the potential association of sleep timing, the gut microbiota, and the metabolites using multiomics data in children living in the northwest region in China. Apparently healthy children, aged 3–8 years, were consecutively recruited from Lanzhou City of Gansu province, located in Northwest China. Excluded from the study were those who had a serious chronic infection, any current infection, or received antibiotic treatment within 30 days before taking part in this study. Signed consent forms were obtained from parents or guardians of the participants.

The information from study participants, collected using a designed questionnaire included age, sex, height, weight, residential address, medication use, defecation, family history, and living habit. In this study, the participants were classified into two groups according to their typical bedtime (early sleep group: before 22:00, late sleep group: after 22:00). In addition, *Z* scores of body mass index (BMI) by age were calculated and further grouped according to the WHO recommendation [10]. In this study, all biological samples (including faeces and blood) and clinical biochemical indicators were collected according to the standard operation procedure at the second affiliated hospital with Lanzhou University in Gansu province. In detail, the biological samples were collected and immediately storage at a −80 °C freezer, and then rapidly transported to the appointed laboratory on dry ice.

### 2.2. DNA Preparation and Metagenomics Sequencing

DNA extraction was performed using the phenol/trichloromethane method after thawing samples on ice. Extracts were then treated with DNase-free RNase, and DNA quality was measured using agarose gel electrophoresis and a Qubit 3.0 fluorimeter (Thermo Fisher, Waltham, MA, USA). After quality control (concentration and integrity testing) of the total DNA, 500 ng of DNA was fragmented by ultrasound using a Covaris E220 (Covaris, Brighton, UK), then selected to 300~700 bp using magnet beads size-selection. The selected DNA fragments were repaired, then ligated with an indexed adaptor. The ligation products were amplified by PCR, hybridized with an exon probe, and captured by streptavidin beads. The captured DNA were amplified again by PCR, and circularized to obtain a single-stranded circular(ssCir) library. The ssCir library was then amplified through rolling circle amplification (RCA) to obtain DNA nanoball(DNB). The DNB was then loaded to a flowcell, and sequenced using a DNBSEQ Platform (BGI-Shenzhen, Shenzhen, China). The raw reads that had 50% low-quality bases (quality ≤ 20) or more than five ambiguous bases were excluded, and the remaining reads were filtered in order to eliminate host DNA, using the reference human genome as described previously [11]. Metagenomics sequencing was performed on the DNBSEQ-T1 platform with pair-end library and read length of 150 bp [12]. Quality control tests were performed, and adaptor and host contamination were filtered using fastp [13] and bowtie2 [14].

### 2.3. Taxonomic and Functional Profiling Acquisition

Taxonomic profiles were generated from high-quality reads using Metaphlan 3.0 [15], (-input_type fastq-ignore_viruses-nproc 6), as described on the company website (MetaPhlAn3-The Huttenhower Lab (harvard.edu). For functional profiling, HUMAnN 3.0 (-i input_clean_data -o output --threads 10 --memory-use maximum --remove-temp-output) was used to efficiently and accurately profile the abundance of microbial metabolic pathways and other molecular functions from the metagenomic sequencing data (clean data) according to the corresponding paper [16] and official website (https://huttenhower.sph.harvard.edu/humann).

### 2.4. Alpha- and Beta-Diversity

Alpha diversity (within-sample diversity, R 4.0.3 vegan: diversity (data, index = ‘richness/shannon/Simpson/InSimpson’)) was calculated using the richness, Shannon index, Simpson index, and Inverse Simpson index, depending on the taxonomic profiles [17]. Beta diversity (between-sample diversity, R 4.0.3 ape: pcoa (‘bray_curtis distance’, correction = “none”, rn = NULL), was calculated using the Bray-Curtis distance depending on the taxonomic profiles. Permutational multivariate analysis of variance [PERMANOVA; code: R 4.0.3:adonis (dist~phe, permutations = 10,000)] was performed, on the gut microbial species/genus abundance profiles to study the effect of group on the gut microbiome.

### 2.5. Metabolomic Detection and Analysis

#### Sample Preparation and Extraction

The biological samples were freeze-dried using a vacuum freeze-dryer (Scientz-100F). The freeze-dried samples were crushed using a mixer mill (MM 400, Retsch, Haan, Germany) with a zirconia bead for 1.5 min at 30 Hz. The lyophilized powder (50 mg) was placed into 1.2 mL of 70% methanol solution and vortexed for 30 s every 30 min for six times in total. After centrifugation at 12,000 rpm for 3 min, the extracts were filtered (SCAA-104, 0.22 μm pore size; ANPEL, Shanghai, China) before UPLC-MS/MS analysis.

### 2.6. UPLC Conditions

The sample extracts were analyzed using an UPLC-ESI-MS/MS system (UPLC, SHIMADZU Nexera X2, Kyoto, Japan; MS, Applied Biosystems 6500 Q TRAP, Thermo Fisher Scientific, Waltham, America). The analytical conditions were as follows: UPLC, column, Agilent SB-C18 (1.8 µm, 2.1 mm × 100 mm); the mobile phase consisted of solvent A, pure water with 0.1% formic acid, and solvent B, acetonitrile with 0.1% formic acid. Sample measurements were performed with a gradient program that employed the starting conditions of 95% A and 5% B. Within 9 min, a linear gradient of 5% A and 95% B was programmed, and a composition of 5% A and 95% B was maintained for 1 min. Subsequently, a composition of 95% A and 5.0% B was adjusted within 1.1 min and kept for 2.9 min. The flow velocity was set to 0.35 mL per minute; The column oven temperature was set to 40 °C, and the injection volume was 2 μL. The effluent was alternatively connected to an ESI-triple quadrupole linear ion trap (QTRAP)-MS system.

### 2.7. ESI-Q TRAP-MS/MS

The ESI source operation parameters were as follows: source temperature, 500 °C; ion spray voltage (IS), 5500 V (positive ion mode)/−4500 V (negative ion mode); ion source gas I (GSI), gas II(GSII), and curtain gas (CUR) were set at 50, 60, and 25 psi, respectively; collision-activated dissociation (CAD) was high. Instrument tuning and mass calibration were performed using 10 and 100 μmol/L polypropylene glycol solutions in the QQQ and LIT modes, respectively. QQQ scans were acquired in MRM experiments with a collision gas (nitrogen) set to the medium. The declustering potential (DP) and collision energy (CE) for individual MRM transitions were determined with further DP and CE optimizations. A specific set of MRM transitions was monitored for each period according to the metabolites eluted within this period.

### 2.8. KEGG Annotation and Enrichment Analysis

Identified metabolites were annotated using the KEGG Compound database (http://www.kegg.jp/kegg/compound/, accessed at any time currently) and annotated metabolites were mapped to the KEGG Pathway database (http://www.kegg.jp/kegg/pathway.html, accessed at any time currently).

### 2.9. Metagenome-Wide Association Study and Metabolomics Analysis

The identified species, genus, phyla, the functional module, and the relative abundance of each sample was compared between the groups of children with early bedtimes and those with late bedtimes using Wilcoxon rank-sum test followed by a Storey’s FDR correction. Tests for correlation between the baterial taxa and the functional modules via semi-partial Spearman’s rank-order correlation tests, adjusted for BMI, gender, and age. Pair-wise comparison of species and functional modules between the groups of children with early or late bedtime was conducted using paired Wilcoxon rank-sum test and partial spearman correlation tests with a multiple testing correction of Benjamini–Hochberg correction. Metabolomics analysis on feces and serum samples were performed as a targeted metabolic analysis framework based on a set of manually curated reference modules developed at an appointed laboratory according to a previous study protocol [18].

### 2.10. Statistical Analysis

Analyses were calculated using the statistical software R, version 4.2.0 (R-Core-Team, 2018) with Bioconductor 3.13 adjusting for all covariates as specified in the previous section [19]. The differences in clinical indicators among the children with early or late bedtime were determined using Student’s *t*-test (code: t.test(data, group)) or Fisher’s exact test (code: fisher.test(data)). Differentially elevated or depleted gut microbes and metabolites were evaluated by Wilcoxon rank sum test and partial correlation test. The connection of microbes to host metabolites was assessed using Spearman’s rank correlation. Differentially enriched KEGG modules of selected metabolic pathways were identified by Wilcoxon rank sum test (code: wilcox.test(data, group). All statistical tests are two-tailed, and a *p* < 0.05 is considered statistically significant. Principle co-ordinate analysis (PCoA) was performed using the statistical function ape comp in R (version 4.3.1, www.r-project.org). Significantly regulated metabolites between the groups were determined by Wilcoxon rank-sum test (*p* < 0.05). Moreover, taxons and metabolites were correlated with group via partial Spearman correlation tests (R package pcor: code: pcor.test (data, group, c (age, BMI, gender)], method = “spearman”)) adjusting for BMI, age, and gender using the multiple testing Benjamini–Hochberg correction (code: p.adjust (*p* value, method = “BH”)).

## 3. Results

### 3.1. Gut Microbiota Characterization for Samples

A total of 88 children aged 3–8 years (early sleep group: 5.5 ± 2.1 years; late sleep group: 5.7 ± 2.3 years) were included in this study (Table 1). Among them, 79.1% and 52.5% of the early- and late-sleep groups were of normal weight, respectively. The percentage of overweight and obese participants was significantly higher among the children with late sleep (*p* = 0.003). Intriguingly, the average age of the parents of the children with early sleep was a little older than that of the late sleep ones, suggesting that parents played a certain role in early sleep of children, but no significant difference was observed (*p* > 0.05).

In the shotgun metagenomic sequencing, an average of 12.02 ± 1.95 Gb raw reads were obtained for each sample. Raw reads that had 50% low-quality bases (quality ≤ 20) or more than five ambiguous bases were excluded and the remaining rate was 99.42 ± 0.16. Then, the remaining reads were filtered in order to eliminate host DNA and an average of 11.78 ± 1.94 Gb clean reads were finally obtained for further taxonomic and functional annotation (Table S1).

**Figure 1:**
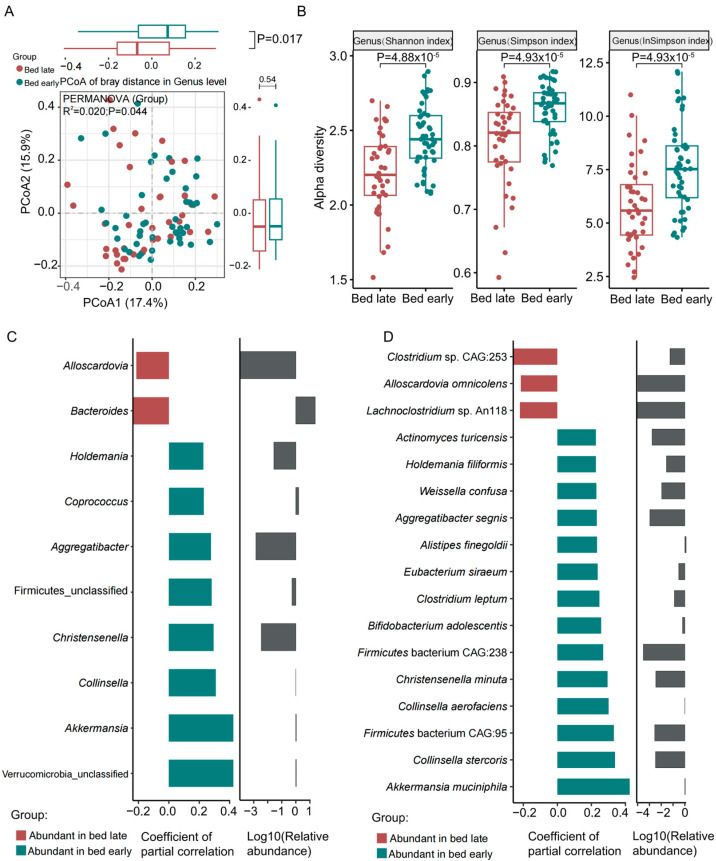
Microbial disorders in the gut of children in bed late and early. (**A**) PCoA showed significant differences of the gut microbiota at genus level. (**B**) The genus alpha diversity based on Shannon index, Simpson index and Inverse Simpson index was significantly different between bed late and early children. (**C**) Significantly different genera and (**D**) species associated with bed early and late. Grey in (**C**,**D**) refers to the logarithm of relative abundance of the corresponding genera and species, the smaller the value, the lower the relative abundance.

PCoA analysis based on the Bray–Curtis distances of genera indicated a significant difference in genera between the two groups (Figure 1A, *p* = 0.017). Alpha diversity based on the Shannon, Simpson, and Inverse Simpson indices showed higher richness and diversity of the gut genera in children with early bedtime (Figure 1B, all *p* < 0.05). At the species level, PCoA showed no significant difference between the two groups (Figure S1A); however, the alpha diversity based on the Shannon index was significantly different between the groups (Figure S1B), suggesting that children with early bedtime have higher richness and diversity of their gut microbiota. Notably, children with early bedtime had significantly higher relative abundance (RA) of *Akkermansia* (*muciniphila*), *Collinsella*, *Christensenella*, *Aggregatibacter*, *Coprococcus*, *Holdemania*, *Collinsella stercoris*, *Firmicutes bacterium* CAG:95, *Collinsella aerofaciens*, *Christensenella minuta*, *Firmicutes bacterium* CAG:238, *Bifidobacterium adolescentis*, *Clostridium leptum*, *Eubacterium siraeum*, *Weissella confusa*, *Holdemania filiformis*, *Actinomyces turicensis*, and *Alistipes finegoldii*, while the children with late bedtime showed higher RA of *Bacteroides* and *Clostridium* sp. CAG_253 (Figure 1C,D). We then analyzed the differences of high abundant phyla, genera, and species (Top 20 abundant genera and species). The RA of Verrucomicrobia was significantly greater among children with early bedtime (Figure S1C). Among the 20 most abundant genera, *Coprococcus*, *Akkermansia*, *Collinsella* were significantly more abundant in children with early bedtime, while Bacteroides were significantly more common among children with late bedtime. Most of the high-abundance genera were *Faecalibacterium*, *Bifidobacterium*, *Roseburia*, *Eubacterium*, *Ruminococcus*, *Blautia*, *Alistipes*, *Anaerostipes*, *Prevotella*, *Fusicatenibacter*, *Streptococcus*, *Parabacteroides*, *Megamonas*, *Lachnospira*, and *Escherichia*; however, their frequencies were not significantly different between the two groups (Figure S1D). The 20 most abundant species were *Faecalibacterium prausnitzii*, *Eubacterium rectale*, *Ruminococcus bromii*, *Roseburia faecis*, *Bacteroides plebeius*, *Alistipes putredinis*, *Roseburia inulinivorans*, *Fusicatenibacter saccharivorans*, *Roseburia intestinalis*, *Eubacterium eligens*, *Blautia wexlerae*, *Prevotella copri*, *Bacteroides vulgatus*, *Bacteroides uniformis*, *Bifidobacterium pseudocatenulatum*, *Bacteroides dorei*, *Bifidobacterium longum*, and *Bacteroides fragilis*, with no significant differences in frequency between the two groups (Figure S1E). It is worth noting that *Bifidobacterium adolescentis* and *Akkermansia muciniphila*, which are probiotic species and *Alistipes finegoldii,* which has been associated with fatty acid activation and utilization [20] were significantly more abundant in guts of early sleep children, and were linked to a number of metabolic pathways in these children (Figure S2A–C). *A. muciniphila* is associated with amino acid biosynthesis, ribonucleotides biosynthesis, coenzyme A biosynthesis, and folate transformations (Figure S2A). *B. adolescentis* is associated with glucosamine biosynthesi, and amino acid synthesis (Figure S2B). *A. finegoldii* is primarily associated with stearate biosynthesis and L-ornithine/L-arginine/L-valine/L-lysine/L-histidine/L-citrulline/L-serine and glycine biosynthesis (Figure S2C). Interestingly, the main pathways of the children with late bedtime were primarily associated with three species: *Klebsiella pneumonia* was associated with assimilatory sulfate reduction, polyamine biosynthesis, and nucleotides biosynthesis; *Clostridium* sp. CAG253 was associated with ribonucleotide de novo biosynthesis and purine ribonucleosides degradation; and *Clostridium disporicum* was associated with aromatic amino acid biosynthesis and chorismate biosynthesis (Figure S2C).

Humann3.0 was used to analyze the metabolic pathways of the gut microbiota (Table S2). A total of 106 pathways were significantly more frequently identified in the microbiomes of children with early bedtime (Figure S3). These pathways included: (1) 5-aminoimidazole ribonucleotide biosynthesis, pyrimidine deoxyribonucleotide phosphorylation, superpathway of purine deoxyribonucleosides degradation, superpathway of pyrimidine nucleobases salvage, adenosine ribonucleotide and guanosine deoxyribonucleotide de novo biosynthesis, superpathway of adenosine/guanosine nucleotides de novo biosynthesis; (2) biosynthesis for aromatic amino acid, branched chain amino acids, L-lysine, L-threonine, L-methionine, L-serine, glycine, L-arginine, L-citrulline, L-histidine, L-isoleucine, L-proline, L-lysine, L-methionine, L-ornithine, L-tryptophan; (3) superpathway of fatty acid biosynthesis initiation, saturated fatty acid elongation, and stearate biosynthesis; (4) stachyose and starch degradation, pyruvate fermentation to acetate and lactate, pentose phosphate pathway, glycogen biosynthesis, peptidoglycan biosynthesis III; (5) others: CDP-diacylglycerol biosynthesis, chorismate biosynthesis, coenzyme A biosynthesis, folate transformations, inosine-5-phosphate biosynthesis, NAD de novo biosynthesis I, UDP-*N*-acetylmuramoyl-pentapeptide biosynthesis, and UMP biosynthesis. In children with late bedtime, only 42 metabolic pathways, including guanosine ribonucleotides de novo biosynthesis, and NAD salvage pathway III (to nicotinamide riboside) were highly abundant (Figure S4). These results suggested that the gut microbial diversity, richness and function were significantly decreased in children with late bedtime.

### 3.2. Differential Analysis of the Gut Microbial Metabolites

**Figure 2:**
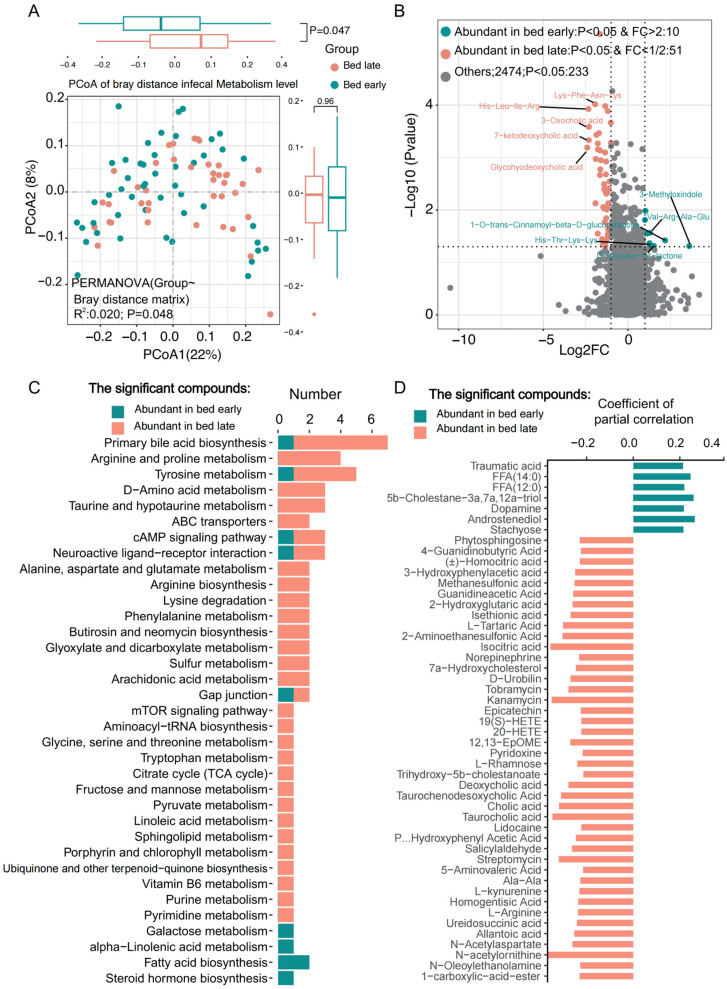
Differential analysis of the gut microbial metabolites between bed early and late children. (**A**) PCoA of the gut microbial metabolites were significantly different between two groups. (**B**) Volcano plot showed the differences of the metabolites, and 10 metabolites were significantly abundant in bed early children while 51 metabolites were obviously enriched in bed late children. (**C**) Enrichment of the KEGG pathways participated by the significantly different microbial metabolites in two groups. (**D**) The significantly different metabolites with KEGG pathway annotation for two groups.

PCoA analysis based on the Bray distance of the gut microbiota metabolites were obviously different between two groups (R^2^ = 0.02, *p* = 0.048), and much more fecal metabolites were observed in children with late bedtime (Figure 2A). In total, 2707 fecal metabolites were detected and 233 metabolites showed significant differences (*p* < 0.05, pcor) in frequency between the two groups, and in which, there were 61 fecal metabolites with fold change greater than 2 or less than 1/2 (Figure 2B). Interestingly, 10 metabolites including traumatic acid, FFA (14:0), FFA (12:0), dopamine, etc. were abundant in children with early bedtime, while 51 metabolites including taurocholic acid, streptomycin, kanamycin, *N*-acetylornithine, isocitric acid, and L-tartaric acid were obviously abundant in children with late bedtime (Figure 2D), indicating that children with late bedtime remain in an active metabolism. Among the functional annotation data of the various metabolites, we found that the metabolites in children with late bedtime were mainly related to primary bile acid biosynthesis, amino acid metabolism, purine and pyrimidine metabolism, fructose and mannose metabolism, TCA cycle, tryptophan metabolism, and neuroactive ligand-receptor interaction; however, in children with early bedtime, galactose metabolism, alpha-linolenic acid metabolism, fatty acid biosynthesis, steroid hormone biosynthesis were the main KEGG pathways involved (Figure 2C).

**Figure 3:**
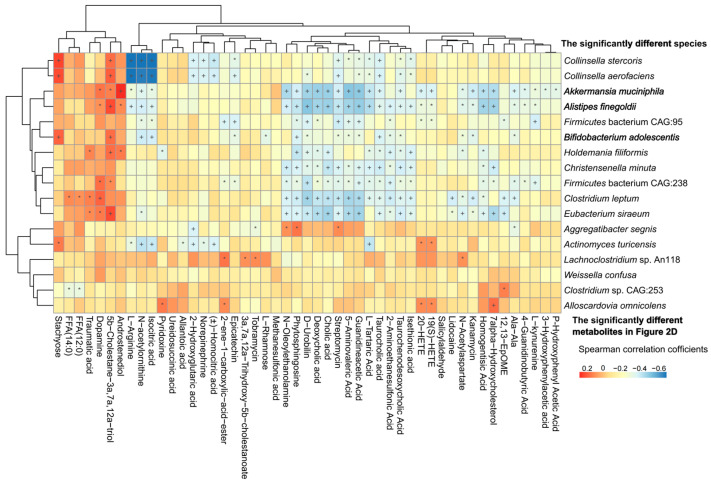
Correlation analysis between the significantly different species and the significantly changed microbial metabolites with KEGG pathways annotated in Figure 2D. +, *p* < 0.01; *, *p* < 0.05.

Correlation analysis between 17 significantly different species and 61 obviously distinct fecal metabolites were analyzed. *Akkermansia muciniphila*, *Bifidobacterium adolescentis*, *Clostridium leptum*, *Eubacterium siraeum*, *Alistipes finegoldii*, *Holdemania filiformis* were significantly positively associated with stachyose, FFA (14:0), FFA (12:0), traumatic acid, dopamine, 5b-Cholestane-3a,7a,12a-triol, and androstenediol, which were highly enriched in children with early bedtime, but were negatively correlated with metabolites highly abundant in children with late bedtime (Figure 3).

### 3.3. Difference Study of the Plasma Metabolites

**Figure 4:**
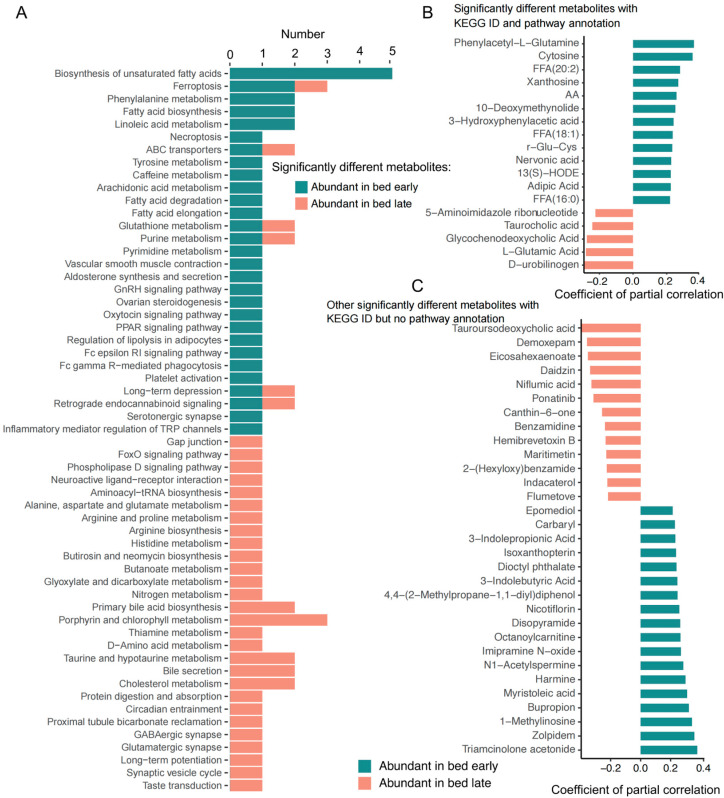
Variation analysis of the plasma metabolites. (**A**) Enrichment of the KEGG pathways involving significantly differential metabolites in plasma between two groups. (**B**) Significantly different metabolites with KEGG ID and pathways annotated between two groups. (**C**) Significantly different metabolites with KEGG ID but no pathways annotated between two groups.

The gut microbial metabolites could be absorbed into blood to influence on host physiology; thus, we detected the plasma metabolites of children with early and with late bedtime. In total, 1538 plasma metabolites were detected and of those, 123 metabolites were significantly different between the two groups. Among children with early bedtime, 77 plasma metabolites were abundant, while 46 plasma metabolites were enriched in children with late bedtime. The KEGG pathways of different metabolites for children with early bedtime included biosynthesis of unsaturated fatty acids, fatty acid biosynthesis, linoleic acid metabolism, arachidonic acid metabolism, fatty acid degradation and elongation, PPAR signaling pathway, and regulation of lipolysis in adipocytes (Figure 4A). However, the metabolites enriched in children with late bedtime were mainly focused on amino acids metabolism, FoxO signaling pathway, primary bile acid biosynthesis, bile secretion, cholesterol metabolism, protein digestion and absorption, and GABAergic synapse and glutamatergic synapse (Figure 4A). Among children with early and with late bedtime, there were 13 and 5 significant metabolites with pathway annotations, and 18 and 13 other significant metabolites with database IDs (Figure 4B,C), respectively.

#### KEGG Pathways Participated by the Significantly Different Metabolites in Plasma

We then analyzed the correlation between the gut microbiota and plasma metabolites. The species with significantly different abundance between the two groups including *Eubacterium siraeum*, *Akkermansia muciniphila*, *Bifidobacterium adolescentis*, *Clostridium leptum*, *Christensenella minuta*, *Firmicutes bacterium* CAG:95, and *Alistipes finegoldii* were obviously associated with plasma metabolites (Figure S5). The gut microbiota, fecal metabolites, and plasma metabolites were further assessed and 8 metabolites were commonly shared (Figure S6A). In common metabolites, 5 metabolites were negatively correlated with bedtime, such as glycoursodeoxycholic acid, glycohyodeoxycholic acid, lle-His-Ala-Glu, taurocholic acid, and carnitine C15:1: DC. (Figure S6B).

## 4. Discussion

Sleep could be beneficial to human health by changing the gut microbiome and affecting the metabolites, particularly in children at the rapid growth stage. Thus, in this study, we investigated the gut microbiota and fecal and plasma metabolites among children with early bedtimes and those with late bedtimes, as well as the differences in these observations between these two groups of children who live in northwest China. The findings of this study showed that late bedtime was closely associated with reduced gut microbial diversity and richness, as well as increased BMI. Furthermore, the composition of fecal and blood metabolites is markedly different among these groups. Bedtime could affect the gut microbiota, fecal and plasma metabolites via the child’s circadian rhythm, synchronous metabolism, or eating behaviors. Therefore, the selection of appropriate bedtime in children should receive attention in its role during the development of health promotion.

Here we observed a negative correlation between late bedtime and higher prevalence of overweight and obesity in children, which was consistent with the previous findings reported that individuals with shorter sleep duration had more risk of being overweight and obese [21,22]. A healthy microbiome is often thought to exhibit robust species richness and evenness, and is theorized to confer a safeguard for health. By contrast, the low species diversity is more associated with gut microbiota dysbiosis [23], which is implicated in various clinical manifestations [24]. Our study reported the alpha-diversity of gut microbiota in children with late bedtime was significantly lower compared to those with early bedtime, which was basically consistent with previous findings [25]. An idea was thus proposed that increased microbial diversity might signify an impetus for an early bedtime habit.

As expected, there were specific groups of commensal gut microbiota in children with early bedtime and in those with late bedtime. The taxa have been associated with sleep-related neurochemicals, which possess the mediating roles of neurotransmitters. As shown in this study (Figure S2), an obvious increase in Verrucomicrobia may bring a significant health benefit through the *A. muciniphila* species, which is a designated gate-keeper of the gut epithelial layer to maintain gut barrier integrity in order to prevent pathogenic and pro-inflammatory bacteria from entering circulation [26]. In contrast, a significant increase in the RA of Bacteroides was discovered in children with late bedtime, which is one of the key taxon producing short-chain fatty acids (SCFAs) via the succinate pathway to enhance sleep efficiency [27]. Similarly, the increase level of *Coprococcus* in children with late bedtime that is known for boosting butyrate production, which possibly serve as a sleep-inducing signal molecule to improve sleep [28,29]. In addition, studies on the effect of *Bifidobacterium* demonstrated its role in regulating mood and improving sleep via the well-established immune-regulating properties [30]. Here, our study had offered an interpretation regarding the effects of bedtime on gut microbial composition. In addition, KEGG functional enrichment analysis and correlation analysis showed the differential changes in the metabolic patterns might be associated with altered gut microbiota due to different bedtime. The underlying reasons might be related to the difference of eating behaviors, the body’s rhythm or the combined actions [31].

Fecal metabolites were distinct between the two study groups; they were more enriched in children with late bedtime, which provided insight into the link between sleep pattern and the gut microbiota. As a reliable measure of microbial activity, fecal metabolome can indirectly reflect the composition of the fecal microbiome. In this study, several metabolites associated with late bedtime are mainly involved in primary bile acid biosynthesis, including glycoursodeoxycholic acid, hyodeoxycholic acid, glycohyodeoxycholic acid, and taurocholic acid, etc. Bile acids are a class of cholesterol derivatives that are generally synthesized in the liver and excreted into the intestinal lumen to assist in the emulsification and absorption of lipid [32]. These could serve as versatile signaling molecules, and further act as anti-inflammatory and immune-regulatory agents in the intestinal tract and the central nervous system by activating the receptors, which play a certain role in the metabolism of glucose, lipid, and cholesterol [33]. Similarly, tryptophan is an essential aromatic amino acid for protein synthesis and also a key precursor to many microbial and host metabolites, which could play the important role in immune modulation and neurotransmission [34]. In addition, the effects of some of the fecal metabolites, such as glycine and ornithine, in enhancing sleep have been reported in previous studies [35,36]. The correlation analysis indicated that several gut microbiota species, such as *A. muciniphila* and *B. adolescentis*, are associated with specific fecal metabolites in children, indicating that a distinct change in fecal microbiome impacts the host physiology via the alteration in the metabolites they produce.

In addition, differential plasma metabolites were discovered between children with early and and those with late bedtime. The higher level of plasma metabolites associated with late bedtime were mainly involved in porphyrin and chlorophyll metabolism, primary bile acid biosynthesis, bile secretion, taurine and hypotaurine metabolism, and cholesterol metabolism; whereas the metabolites associated with early bedtime were mainly involved in biosynthesis of unsaturated and saturated fatty acids, phenylalanine metabolism, and linoleic acid metabolism. The relationship between sleep timing and the metabolites are thought to mainly derive from their roles in neuro-immunoendocrine regulation, and are affected by multiple factors, such diets and living environment. In line with previous studies [37,38], our study further examined the relationship of the gut microbiome with the metabolome. Thus, the complicated microbe-metabolite associations might indicate that the specific gut microbial species produce that metabolites, and the metabolites could promote or inhibit the growth of the gut microbial species.

## 5. Conclusions

In conclusion, the current study had characterized the gut microbial composition and function, and revealed differences in metabolites from fecal and plasma in children with early or late bedtime, based on multi-omics data, suggesting that late bedtime could decrease the diversity, richness and function of the gut microbiota, and increase unnecessary gut microbial metabolites, which might affect the physical and mental development in children.

## Figures and Tables

**Table 1:** Baseline characteristics for included children in the northwest region of China.

Variables	Early Bedtime	Late Bedtime	*p* Value
N	48	40	−
Age	5.5 ± 2.1	5.7 ± 2.3	0.685
Sex, % male	43.8	52.5	0.399
Current age of father	37.0 ± 4.5	36.3 ± 5.4	0.517
Current age of mother	34.8 ± 4.1	34.0 ± 4.6	0.393
Height	113 (106–125)	110 (100–117)	
Weight	20 (17–30)	20 (15.5–30)	
BMI status			0.003
Thin (n, %)	4 (8.3)	1 (2.5)	
Normal (n, %)	38 (79.1)	21 (52.5)	
Overweight (n, %)	3 (6.3)	5 (12.5)	
Obesity (n, %)	3 (6.3)	13 (32.5)	

## Data Availability

The data that support the findings of this study are openly available at the China National GeneBank Database (CNGBdb) with accession number of CNP0004326.

## Supplementary Materials

The following supporting information can be downloaded at: https://www.mdpi.com/article/10.3390/nu15204315/s1, Figure S1: The gut microbial diversity was significantly different at species level (Figure S1A: beta diversity; Figure S1B: alpha diversity), and the top abundant phyla (Figure S1C), genera (Figure S1D) and species (Figure S1E) were showed; Figure S2: KEGG pathways participated by *Akkermansia muciniphila*, *Bifidobacterium adolescents*, and *Alistipes finegoldii*; Figure S3: Microbial metabolic pathways highly enriched in children with bed early; Figure S4: Microbial metabolic pathways highly enriched in children with bed late; Figure S5: Correlation analysis between the significantly different metabolites in plasma and the significantly different species in two groups; Figure S6: Venn analysis of the significantly different metabolites in plasma and feces; Table S1: Details of the sequencing data for each sample; Table S2: Microbial metabolic pathways analysis; Table S3: Data corresponding to figures.
