# Supplementary figures and images for: Multiomics Revealed the Multi-Dimensional Effects of Late Sleep on Gut Microbiota and Metabolites in Children in Northwest China
Source: Nutrients. 2023 Oct 10;15(20):4315. doi: 10.3390/nu15204315 (PMC10609417; doi:10.3390/nu15204315)

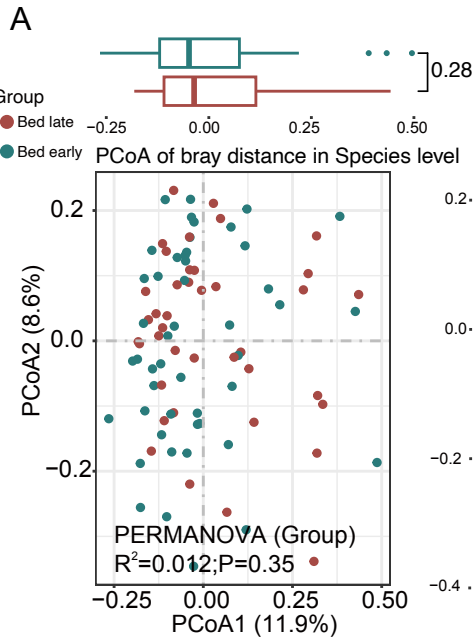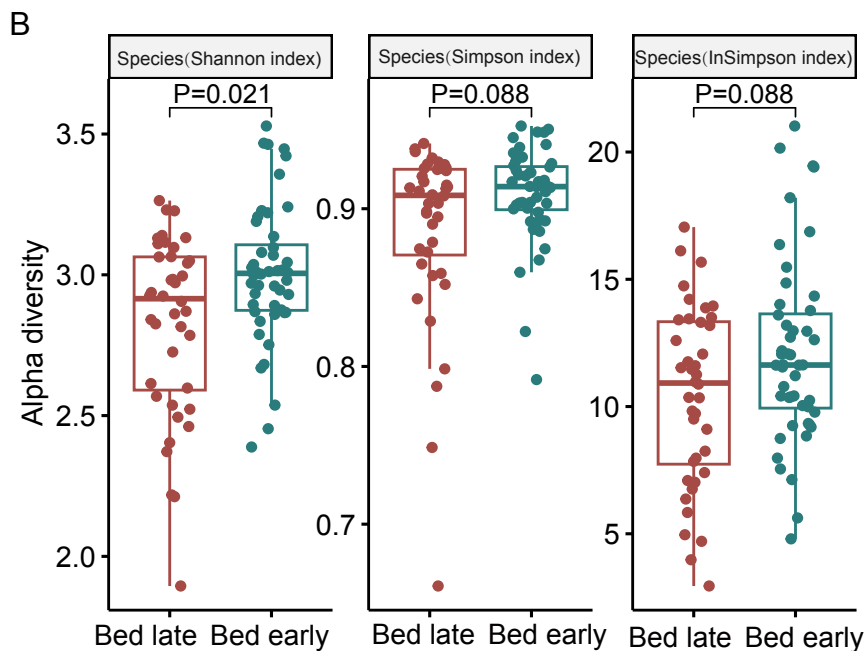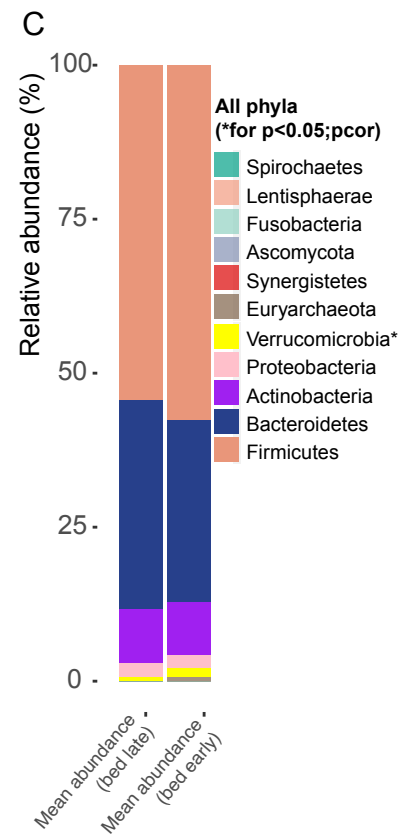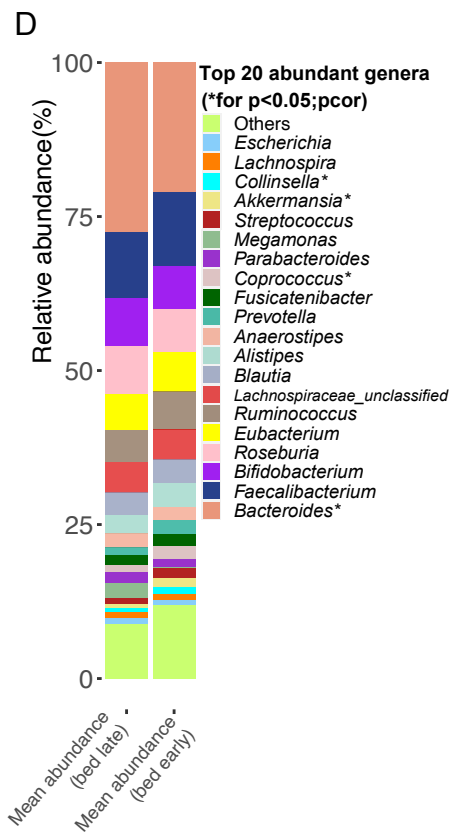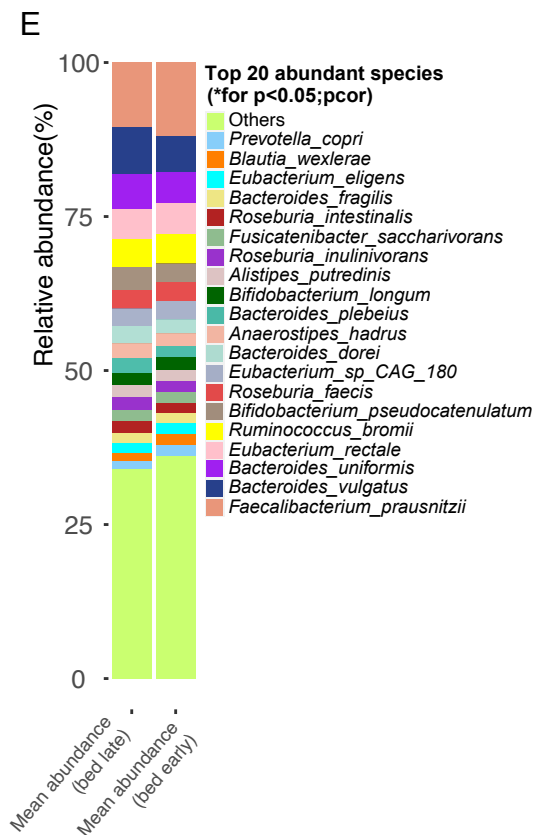

Supplement: Supplementary file 1 [file nutrients-15-04315-s001.zip › Figure S1.pdf]

A

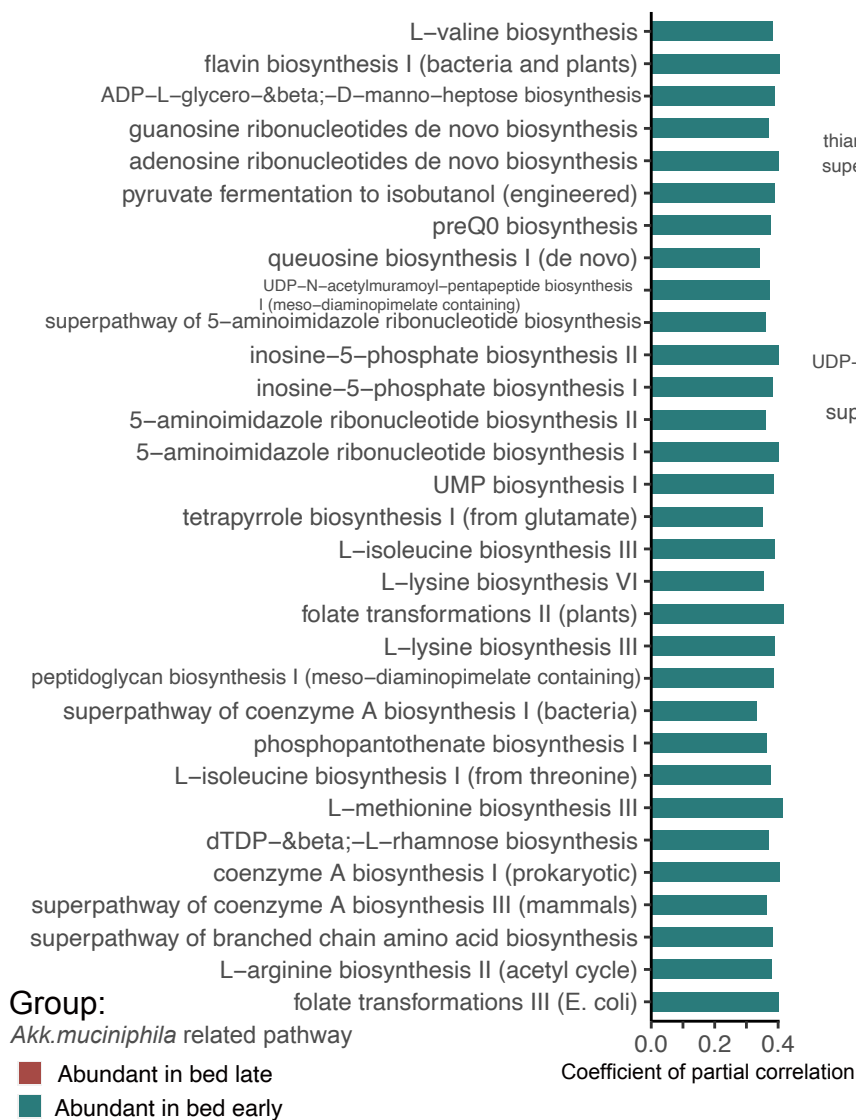

B

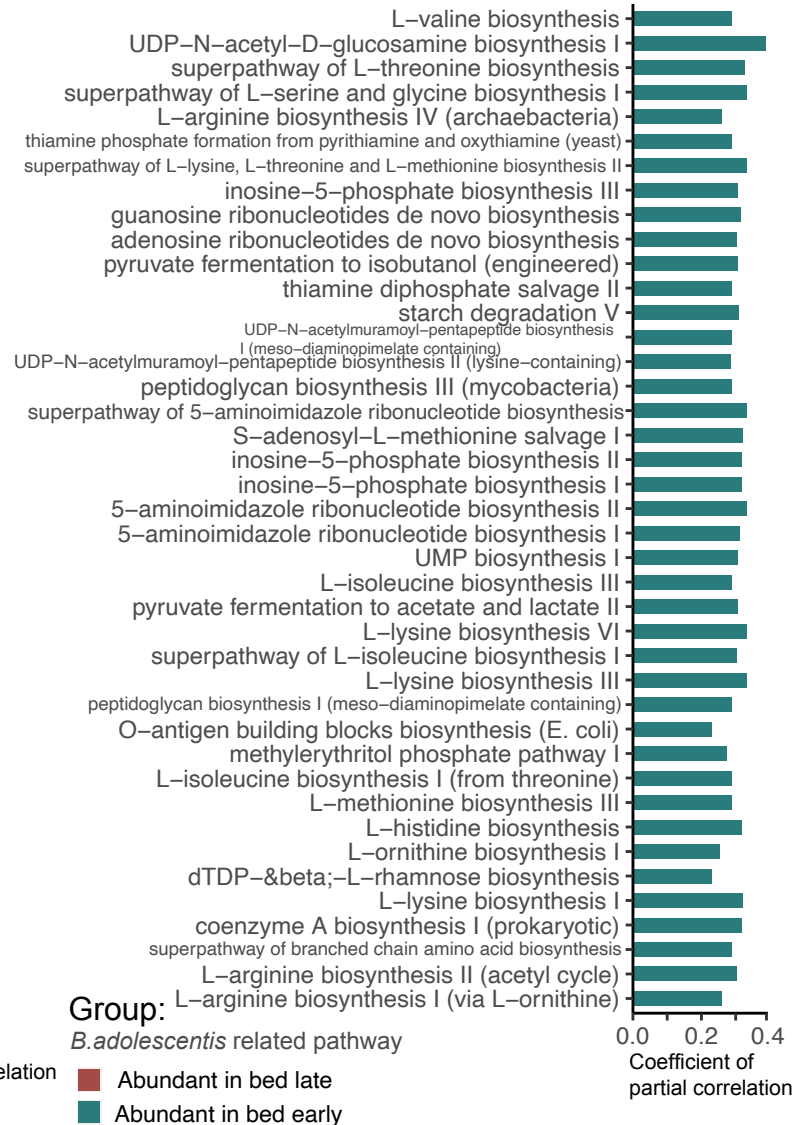

C

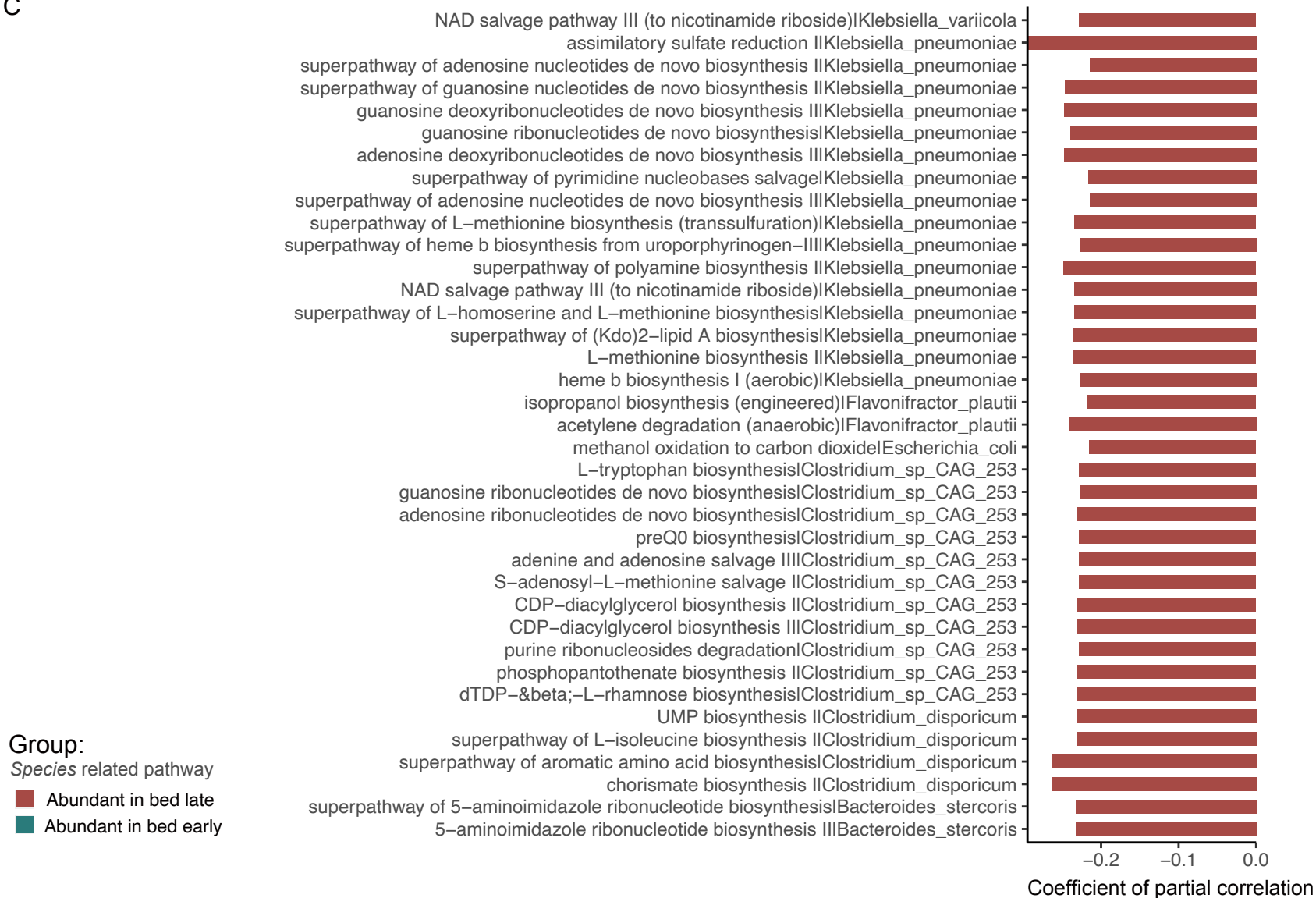

Supplement: Supplementary file 1 [file nutrients-15-04315-s001.zip › Figure S2.pdf]

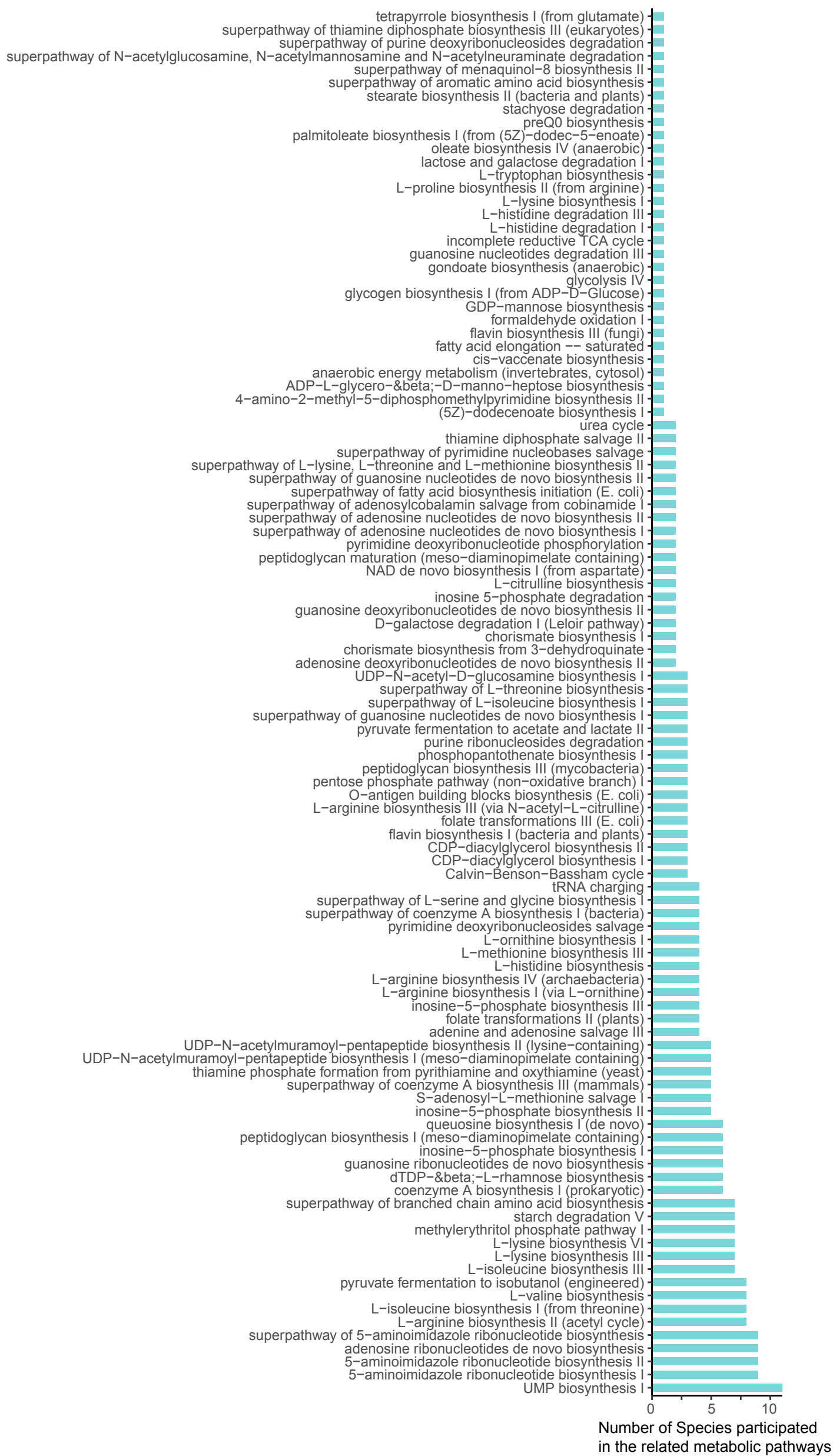

Supplement: Supplementary file 1 [file nutrients-15-04315-s001.zip › Figure S3.pdf]

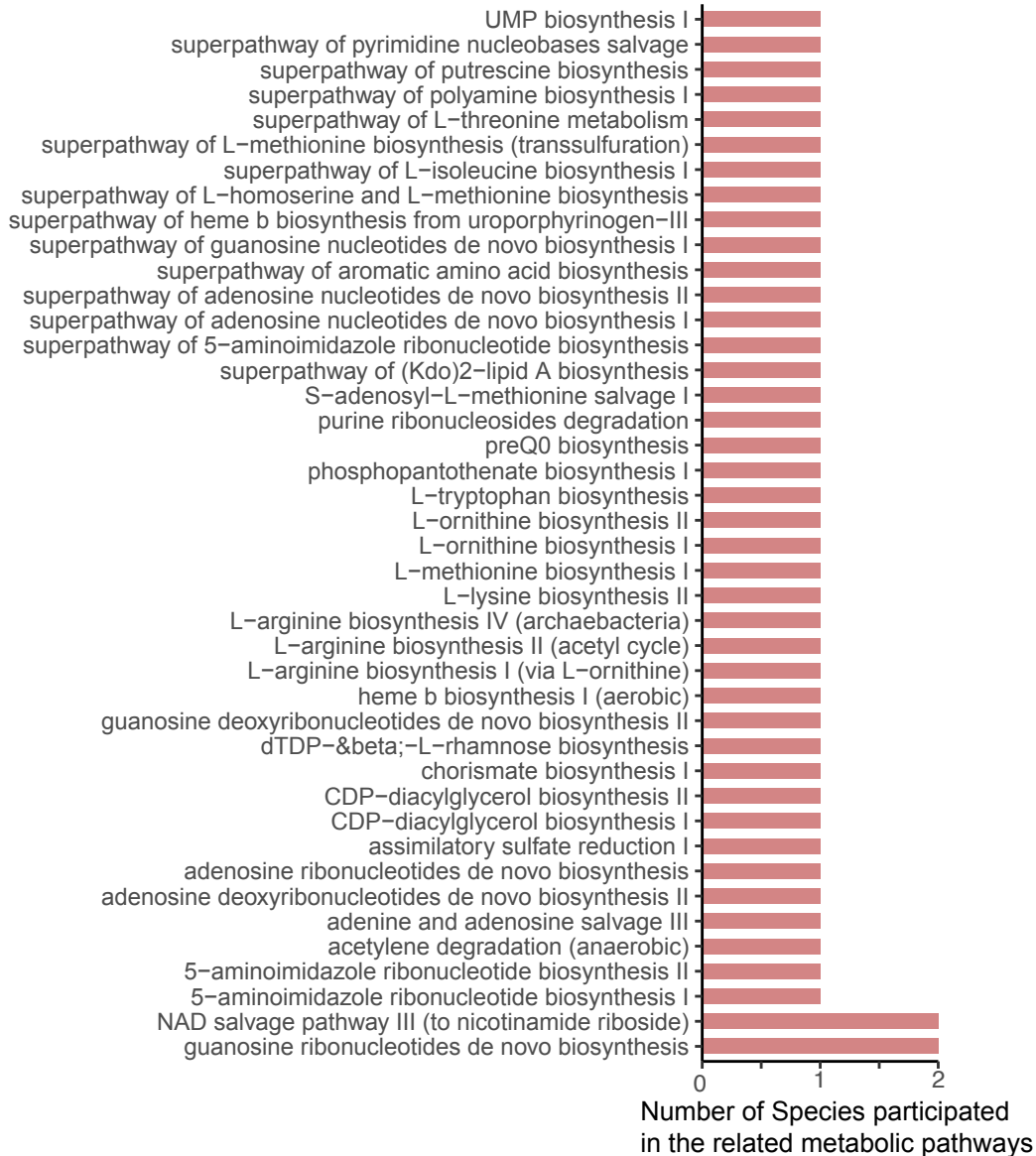

Supplement: Supplementary file 1 [file nutrients-15-04315-s001.zip › Figure S4.pdf]

A

Significant metabolism(Blood)

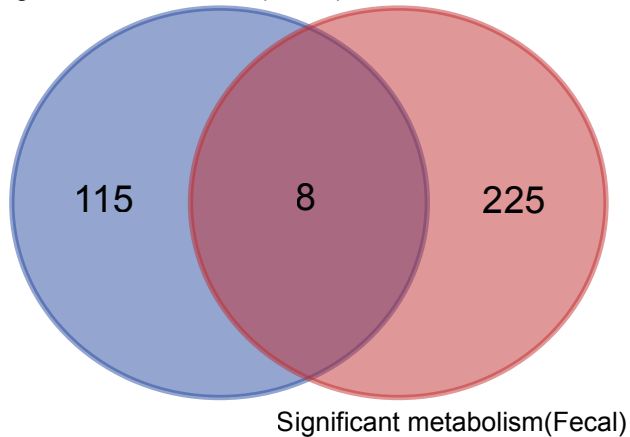

B

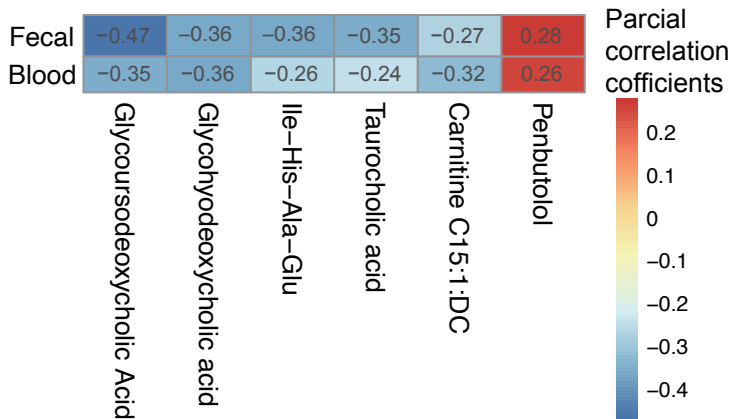

Supplement: Supplementary file 1 [file nutrients-15-04315-s001.zip › Figure S6.pdf]
