# Supplementary material for: Multiomics Revealed the Multi-Dimensional Effects of Late Sleep on Gut Microbiota and Metabolites in Children in Northwest China
Source: Nutrients. 2023 Oct 10;15(20):4315. doi: 10.3390/nu15204315 (PMC10609417; doi:10.3390/nu15204315)
Supplement: Supplementary file 1 [file Figure S5.pdf › Figure S5.pdf]

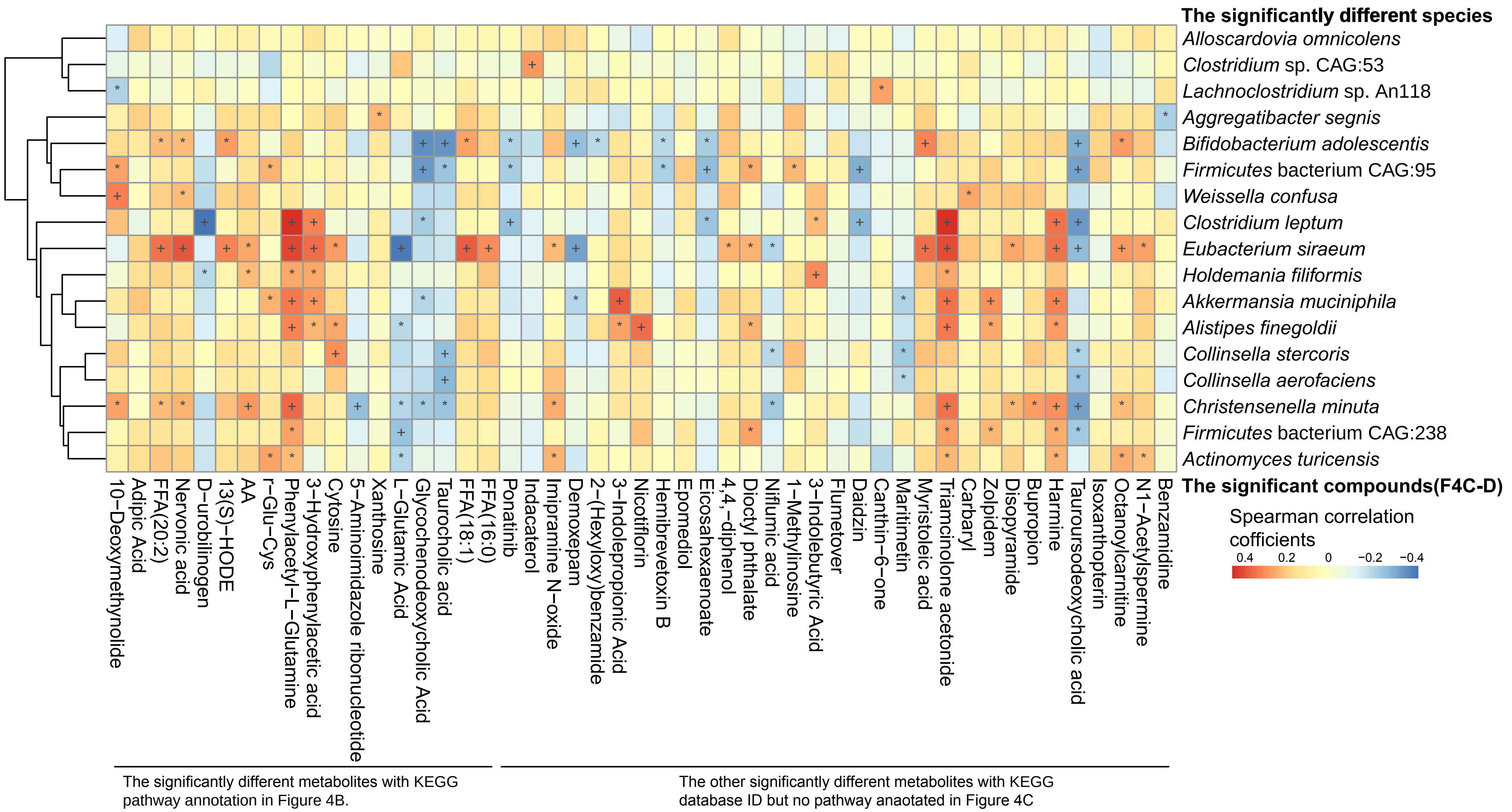
The significantly different species
Alloscardovia omnicolens
Clostridium sp. CAG:53
Lachnoclostridium sp. An118
Aggregatibacter segnis
Bifidobacterium adolescentis
Firmicutes bacterium CAG:95
Weissella confusa
Clostridium leptum
Eubacterium siraeum
Holdemania filiformis
Akkermansia muciniphila
Alistipes finegoldii
Collinsella stercoris
Collinsella aerofaciens
Christensenella minuta
Firmicutes bacterium CAG:238
Actinomyces turicensis
The significant compounds(F4C-D)
10−Deoxymethynolide
Adipic Acid
FFA(20:2)
Nervonic acid
D−urobilinogen
13(S)−HODE
AA
r−Glu−Cys
Phenylacetyl−L−Glutamine
3−Hydroxyphenylacetic acid
Cytosine
5−Aminoimidazole ribonucleotide
Xanthosine
L−Glutamic Acid
Glycochenodeoxycholic Acid
Taurocholic acid
FFA(18:1)
FFA(16:0)
Ponatinib
Indacaterol
Imipramine N−oxide
Demoxepam
2−(Hexyloxy)benzamide
3−Indolepropionic Acid
Nicotiflorin
Hemibrevetoxin B
Epomediol
Eicosahexaenoate
4,4,−diphenol
Dioctyl phthalate
Niflumic acid
1−Methylinosine
3−Indolebutyric Acid
Flumetover
Daidzin
Canthin−6−one
Maritimetin
Myristoleic acid
Triamcinolone acetonide
Carbaryl
Zolpidem
Disopyramide
Bupropion
Harmine
Tauroursodeoxycholic acid
Isoxanthopterin
Octanoylcarnitine
N1−Acetylspermine
Benzamidine
Spearman correlation cofficients
0.4
0.2
0
−0.2
−0.4
The significantly different metabolites with KEGG pathway annotation in Figure 4B.
The other significantly different metabolites with KEGG database ID but no pathway anaotated in Figure 4C
